# Healthcare-delivered lifestyle recommendations and their association with health behaviors in Iran

**DOI:** 10.1371/journal.pone.0345676

**Published:** 2026-11-13

**Authors:** Hanye Sohrabi, Mina Mirzad, Ali Golestani, Sina Azadnajafabad, Naser Ahmadi, Arian Afzalian, Nazila Rezaei, Mohammad-Mahdi Rashidi, Erfan Ghasemi, Negar Rezaei, Moein Yoosefi, Ameneh Kazemi, Shirin Djalalinia, Yosef Farzi, Rosa Haghshenas, Maryam Nasserinejad, Elmira Foroutan Mehr, Sahar Mohammadi Fateh, Farshad Farzadfar

**Affiliations:** 1 Non-Communicable Diseases Research Center, Endocrinology and Metabolism Population Sciences Institute, Tehran University of Medical Sciences, Tehran, Iran; 2 Endocrinology and Metabolism Research Center, Endocrinology and Metabolism Clinical Sciences Institute, Tehran University of Medical Sciences, Tehran, Iran; 3 Development of Research and Technology Center, Deputy of Research and Technology Ministry of Health and Medical Education, Tehran, Iran; Xi’an Jiaotong University, CHINA

## Abstract

**Background:**

Non-communicable diseases (NCDs) are the leading cause of mortality in Iran, driven largely by modifiable lifestyle risk factors. Healthcare providers play a pivotal role in delivering preventive lifestyle recommendations, yet the extent and effectiveness of these efforts remain unclear. This study aimed to assess the distribution of lifestyle counseling across sociodemographic and clinical subgroups and its association with health behavior status in Iran using data from the 2021 WHO STEPS survey.

**Methods:**

We conducted a cross-sectional analysis of 27,704 included adults participating in the nationally representative 2021 STEPS survey in Iran. Data on delivery of ten specific lifestyle recommendations within the past 12 months, covering diet, physical activity, weight management, and tobacco non-initiation/cessation, were collected. Associations between recommendation delivery and health behaviors were analyzed using logistic regression models.

**Results:**

Only 33.4% (95% Confidence Interval (CI): 32.8–34.0) of participants received all ten lifestyle recommendations, while 10.7% (10.2–11.1) received no recommendations. Recommendations were more frequently delivered to females, rural residents, and individuals with multiple comorbidities, indicating a risk-based response by healthcare providers. Notably, tobacco non-initiation/cessation advice was not more commonly offered to patients with multiple chronic conditions. Delivery of lifestyle counseling was associated with positive behavior status: individuals who received weight loss/ maintaining normal weight and physical activity advice were more likely to engage in sufficient physical activity (adjusted odds ratio 1.21(1.13–1.29)), and among current smokers, receiving tobacco cessation recommendation was associated with a higher likelihood of attempting tobacco cessation (aOR: 1.83(1.49–2.24)). A dose-response relationship was observed between the number of nutritional recommendations received and better diet quality (aOR for 6 vs. ≤ 2 recommendations: 1.32 (1.24–1.41)). Geographical disparities were evident, with eastern provinces receiving the least comprehensive counseling.

**Conclusion:**

Delivery of lifestyle recommendations by healthcare providers in Iran shows variation by sociodemographic and clinical factors and is positively associated with behavior status. Given the cross-sectional design, causal or temporal relationships cannot be inferred. These findings suggest opportunities for more equitable and consistent delivery of preventive counseling within national NCD prevention efforts.

## Introduction

Non-communicable diseases (NCDs) represent a major global health challenge, responsible for an estimated 43 million deaths worldwide in 2021, according to the World Health Organization (WHO). Four major NCDs, cardiovascular diseases, cancers, chronic respiratory diseases, and diabetes, account for nearly 80% of all NCD-related deaths globally, with 82% of these occurring in low- and middle-income countries (LMICs) [1]. These conditions are closely linked to modifiable lifestyle risk factors, including poor nutrition, physical inactivity, excess body weight, tobacco use, and harmful alcohol consumption, which necessitate targeted and evidence-based intervention programs [2].

In Iran, a middle-income country, NCDs account for approximately 86% of all deaths, placing a substantial burden on the healthcare system and impeding broader socio-economic development [3]. Most of the leading NCD risk factors in Iran are either behavioral, such as smoking and dietary habits, or metabolic, including hypertension, obesity, hyperglycemia, and dyslipidemia [4]. Reducing the population-level burden of these risk factors is the key to diminishing the rising impact of NCDs.

Healthcare providers across the continuum of care, from primary care physicians to specialists and interprofessional teams, are integral to health promotion and disease prevention by delivering lifestyle recommendations as part of routine clinical practice [5,6]. While preventive counseling has been emphasized in international guidelines for cardiovascular disease, diabetes, chronic respiratory disease, and cancer [7–11], there remains limited evidence on how effectively such recommendations are delivered in practice, particularly in low- and middle-income countries. In Iran, several national strategies have been developed to operationalize this role within the health care system. Aligned with the WHO Global Action Plan for the Prevention and Control of NCDs [12], the Iranian Non‑Communicable Diseases Committee (INCDC) was established through multisectoral collaboration and launched a national action plan in 2015 [13,14]. Despite these policy-level efforts, little is known about how lifestyle recommendations are distributed across the population and whether their receipt translates into measurable behavior change. The WHO STEPwise approach to Surveillance (STEPS) provides a standardized method for collecting nationally representative data on NCDs and their risk factors [15].

Using data from the 2021 STEPS survey [16], this study aims to examine the extent to which lifestyle recommendations are delivered by healthcare providers during routine medical encounters, and to evaluate whether receiving such advice is associated with positive behavior change among Iranian adults. We hypothesized that the delivery of lifestyle recommendations would be higher among individuals with greater comorbidity burden and established risk factors. We further hypothesized that receipt of lifestyle recommendations would be positively associated with healthier behaviors. By addressing this knowledge gap, the study offers important insights into the effectiveness of current NCD prevention strategies and identifies areas for improvement within Iran’s healthcare system.

## Methods

### Study design and participants

This research utilized the data from the STEPS 2021 study [15]. The STEPS 2021 dataset was accessed on October 26, 2024 (10/26/2024). The survey was conducted using a standardized three-step methodology consisting of questionnaire-based interviews, physical measurements, and laboratory assessments among Iranian adults.

A systematic cluster random sampling design was used across all 31 provinces of Iran. The number of clusters allocated to each province was determined using a proportion-to-population size method to ensure representativeness at both national and subnational levels. A total of 3,176 clusters were selected, with 9–10 participants per cluster. In total, 27,874 individuals completed the questionnaire (Step 1), 27,745 underwent physical measurements (Step 2), and 18,119 participants aged 25 years and older provided biological samples for laboratory testing (Step 3).

Only Iranian adults aged 18 years and older were eligible, and the presence of any mental or physical conditions that prevented participation in the study steps and pregnancy were considered exclusion criteria. For the purpose of this study, only data obtained from the questionnaire component were analyzed. We conducted a secondary analysis of the STEPS 2021 dataset and excluded 170 individuals due to missing responses for the lifestyle recommendation questions.

### Data collection

In the first step of the STEPS survey, information was gathered through a standardized questionnaire that addressed a wide range of topics, including demographic characteristics, dietary habits, medical history, physical activity, lifestyle recommendation, tobacco and alcohol consumption, and household assets. The second step involved collecting physical measurements including height, weight, waist, hip circumference, and blood pressure. Height was measured with a standard stadiometer, with participants standing straight against a wall, ensuring contact at the heels, hips, and back of the head. Body weight was measured using a calibrated Inofit digital scale, with participants dressed in light clothing and barefoot. Prior to recording blood pressure, participants were seated and rested for 15 minutes. Blood pressure was then measured three times at three-minute intervals using calibrated Beurer sphygmomanometer, with the final value being the average of the second and third readings. The third step involved serum and spot urine samples. Biological samples were preserved and transported under cold chain conditions to a central laboratory for further analysis.

### Definition of variables

The main outcome of this study was the receipt of ten lifestyle recommendations from physicians or other healthcare providers within the past 12 months. Participants self-reported whether they had received recommendations on the following: tobacco non-initiation/cessation, reducing salt intake, daily consumption of fruits and vegetables, reducing red meat and processed foods, lowering fat intake, increasing fish consumption, consumption of whole grain bread and complex carbohydrates, reducing sugar intake, exercising, and losing weight/maintaining normal weight. Based on the number of recommendations received, participants were categorized into four groups: those who received all ten recommendations, those who received six to nine, those who received one to five, and those who did not receive any recommendation.

This study assessed multiple demographics, behavioral, and clinical variables using the 2021 WHO STEPS survey questionnaire and associated measurements. Participants were categorized into three age groups: 18–39 years, 40–59 years, and 60 years or older. Area of residence was classified as either urban or rural, and marital status was grouped into married, divorced or widowed, and never married. Educational attainment was categorized into three groups: illiterate or 1–6 years of schooling, 7–11 years, and 12 or more years of education. Employment status was recorded as employed, retired, unemployed, or unpaid. The ‘unpaid work’ category was derived from the STEPS 2021 questionnaire and encompasses individuals who engage in activities without receiving regular monetary compensation, including homemakers, individuals assisting with family or household work, students, and military conscripts. Socioeconomic status was measured using the wealth index, which was calculated from 36 household asset-related questions using principal component analysis (PCA). The first principal component, which captured the greatest variation among variables, was selected as the summary measure and categorized into five quintiles, ranging from the poorest (first quintile) to the wealthiest (fifth quintile) segments of the population, in accordance with the STEPS 2021 protocol [16]. Health insurance coverage was recorded as basic only, basic plus complementary, or no insurance. In Iran, the majority of the population is covered by one of several basic health insurance schemes, which provide coverage for a defined package of essential healthcare services and partially reimburse outpatient and inpatient care costs. Because basic insurance does not fully cover all healthcare expenditures, many individuals additionally purchase complementary (supplementary) insurance to cover copayments, coinsurance, and services not fully reimbursed by the basic insurance package. Therefore, participants classified as having “basic plus complementary” insurance were covered by both a basic health insurance scheme and an additional supplementary insurance plan [17,18].

The presence of chronic conditions, including diabetes, hypertension, and hypercholesterolemia was based on self-reported ever diagnoses by healthcare providers. This approach allowed us to focus on individuals aware of their diagnosis. Other comorbidities were similarly defined based on self-report. Participants were considered to have cardiovascular disease (CVD) if they reported ever being told by a healthcare worker that they had a heart attack, angina, or stroke. For chronic respiratory disease, participants were classified as having the condition if they have ever been diagnosed with asthma or chronic obstructive pulmonary disease (COPD), based on self-report of long-term respiratory symptoms and physician diagnosis. Cancer status was defined by the self-report of diagnosis of any types of cancer in the past 12 months. The number of comorbidities was calculated as the count of the following conditions: hypertension, diabetes, hypercholesterolemia, CVDs, chronic respiratory disease, and cancer. We then categorized participants into three groups: 0, 1, or ≥2 comorbidities.

BMI was calculated as weight in kilograms divided by height in meters squared (kg/m²). Participants were classified as underweight or normal weight (BMI < 25 kg/m²), overweight (BMI 25 to <30 kg/m²), or obese (≥30 kg/m²). Physical activity was assessed using the Global Physical Activity Questionnaire (GPAQ). Based on total weekly metabolic equivalent of task (MET), individuals with <600 MET-minutes/week were considered to have insufficient physical activity, per WHO guidelines [19].

Regarding tobacco and alcohol consumption, current tobacco use was defined as any occasional or daily use of tobacco products during the past year. Individuals who reported ever using tobacco but had not used it in the past year were classified as past smokers. Passive smokers were identified as those who reported exposure to secondhand smoke in home or workplace. Participants who reported no history of tobacco use and no exposure to secondhand smoke were considered never smokers. For alcohol consumption, individuals were classified as current drinkers if they had consumed alcohol within the past 12 months, as past drinkers if they had previously consumed alcohol but not within the past year, and as never drinkers if they had never consumed alcohol.

Nutritional practices were evaluated using six key dietary components: intake of fruits, vegetables, red meat, fish, whole grains, and fats. Each component was scored from 0 to 1 based on participants’ responses on the serving/frequency of consumption, reflecting alignment with dietary guidelines and expert recommendations (S1 Table). These scores were then summed to calculate a composite nutrition score with possible values ranging from 0 to 6, where higher scores indicate better dietary quality. Salt intake was assessed using an indirect estimate of daily salt consumption derived from urinary sodium concentration measured in spot urine samples collected during the laboratory phase of the STEPS 2021 survey, applying the Tanaka equation as previously described and validated in the national STEPS 2021 salt intake study [20,21]. Salt intake was classified as appropriate or inappropriate based on the World Health Organization recommendation of less than 5 g of salt per day [22].

### Statistical analysis

Descriptive statistics were used to summarize the characteristics of the study population. To achieve both national and provincial representativeness, a four-step weighting procedure was applied: (1) adjustment for overall non-response, (2) adjusting for non-response specific to each survey step, (3) aligning the sample distribution with provincial population structures by age, sex, and area of residence, and (4) integrating all adjustments into a final composite weight [23]. Categorical variables were reported as weighted prevalence with 95% confidence intervals (CIs). To assess the associations between receiving different lifestyle modification recommendations and various outcomes, logistic regression was used for binary response variables, and ordinal logistic regression was used for the ordinal nutrition score outcome, assuming proportional odds. For each outcome, three models were constructed: a crude (unadjusted) model, an age- and sex-adjusted model (Model II), and a fully adjusted multivariate model (Model III). In the fully adjusted models, covariates were selected using backward stepwise logistic regression with a retention threshold of p < 0.20, performed separately for each outcome (see Table 4 footnotes). Statistical significance was determined at a p-value threshold of <0.05. All analyses were conducted using R 4.4.1, and the ‘survey’ package was used to account for the complex survey design.

### Ethics approval and consent to participate

Ethical approval for this study was obtained from the Research Ethics Committee of the Endocrine & Metabolism Research Institute, Tehran University of Medical Sciences (Approval ID: IR.TUMS.EMRI.REC.1403.056; Approval date: 05 August 2024). The analysis was conducted using anonymized data from the 2021 WHO STEPS survey. The STEPS survey itself received ethical approval from the relevant national ethics committee, and all participants provided informed consent prior to participation. No identifiable personal information was accessed by the authors.

## Results

### Population characteristics

Among 27,704 participants, 44.7% were males and 75.0% lived in urban areas, with roughly even proportions in the 18–39 and 40–59 age groups (Table 1a). Most participants were married (76.6%), 37.0% were employed and nearly half (48.5%) were unpaid workers. Obesity (25.0%) and insufficient physical activity (50.5%) were common, while current tobacco and alcohol use were reported by 14.0% and 3.8% of participants, respectively. Full sociodemographic characteristics are presented in Table 1a, and behavioral, and clinical characteristics in Table 1b.

**Table 1:** (a) Baseline sociodemographic characteristics of the study population. (b) Baseline behavioral and clinical characteristics of the study population.

Variable		Count (n)	Percentage (95% CI)
a			
Sex	Female	15291	55.33 (54.67-55.98)
	Male	12413	44.67 (44.02-45.33)
Age Group	18-39 years old	11093	39.44 (38.8-40.08)
	40-59 years old	10700	39.15 (38.5-39.79)
	≥ 60 years old	5911	21.41 (20.87-21.95)
Residential Area	Rural	7565	25.04 (24.48-25.59)
	Urban	20139	74.96 (74.41-75.52)
Marriage Status	Never Married	4213	15 (14.54-15.47)
	Married	21239	76.64 (76.08-77.19)
	Divorced/Widowed	2252	8.36 (7.99-8.72)
Years of Schooling	illiterate/1-6	10704	37.27 (36.64-37.91)
	7-11	5168	19.12 (18.6-19.64)
	≥12	11640	43.61 (42.95-44.27)
Employment Status	Unemployed	1539	5.27 (4.98-5.56)
	Unpaid Job	13418	48.45 (47.79-49.11)
	Employed	10035	36.98 (36.34-37.61)
	Retired	2520	9.31 (8.92-9.69)
Wealth Index	1^st^ quantile (poorest)	5243	19.02 (18.5-19.54)
	2^nd^ quantile	5234	20.63 (20.08-21.18)
	3^rd^ quantile	5243	18.95 (18.43-19.47)
	4^th^ quantile	5242	19.99 (19.45-20.53)
	5^th^ quantile (richest)	5235	21.41 (20.85-21.97)
Insurance Status	None	2524	9.77 (9.37-10.17)
	Basic	17319	61.86 (61.22-62.51)
	Basic + Complementary	7545	28.37 (27.77-28.97)
	1	5892	21.3 (20.76-21.84)
	2 or more	4615	16.71 (16.22-17.21)
b			
BMI	Normal/Underweight	10346	36.98 (36.35-37.61)
	Overweight	10430	38.04 (37.4-38.68)
	Obese	6798	24.98 (24.41-25.55)
Physical Activity	Appropriate	12732	49.47 (48.78-50.16)
	Inappropriate	12536	50.53 (49.84-51.22)
Tobacco Use	Never Smoker	15591	56.75 (56.09-57.4)
	Passive Smoker	6817	23.82 (23.26-24.37)
	Past Smoker	1479	5.42 (5.12-5.72)
	Current Smoker	3785	14.01 (13.55-14.47)
Alcohol Use	Never Drinker	25685	93.14 (92.8-93.48)
	Past Drinker	788	3.02 (2.8-3.25)
	Current Drinker	974	3.84 (3.58-4.1)
Diabetes Mellitus	No	24819	89.46 (89.06-89.87)
	Yes	2876	10.54 (10.13-10.94)
Hypertension	No	21618	78.22 (77.68-78.76)
	Yes	6078	21.78 (21.24-22.32)
Hypercholesterolemia	No	22788	82.18 (81.68-82.69)
	Yes	4903	17.82 (17.31-18.32)
Cardiovascular Disease	No	25653	92.58 (92.24-92.93)
	Yes	2033	7.42 (7.07-7.76)
COPD/ Asthma	No	26422	95.61 (95.34-95.88)
	Yes	1243	4.39 (4.12-4.66)
Cancer	No	27333	98.8 (98.66-98.95)
	Yes	332	1.2 (1.05-1.34)
Comorbidities	0	17128	61.99 (61.35-62.63)
	1	5892	21.3 (20.76-21.84)
	2 or more	4615	16.71 (16.22-17.21)

### Distribution of lifestyle recommendations across sociodemographic groups

Table 2 presents the distribution of total lifestyle recommendations received across sociodemographic and behavioral subgroups; the prevalence of individual recommendation types by subgroup is provided in S2 Table. Overall, 33.4% (32.8–34.0) of participant received all ten recommendations, 35.8% (35.2–36.5) received six to nine, 20.1% (19.6–20.7) received one to five, and 10.65% (10.2–11.1) received none. A provincial comparison showed the lowest coverage of all-ten delivery in eastern provinces and the highest in the northwest, with approximately inverse pattern for receiving no recommendations (Fig 1). Among nutritional recommendations, advice to increase fruit and vegetable consumption was most frequently delivered (77.0%, 76.4–77.6), while salt-reduction advice was less common (71.0%, 70.4–71.6). Among behavioral recommendations, physical activity advice was most common (72.3%, 71.7–72.9) and weight-loss/weight-maintenance advice were less delivered (69.1%, 68.5–69.7); tobacco non-initiation/cessation advice was the least commonly reported overall (49.8%, 49.2–50.5)

**Table 2:** Distribution of total lifestyle recommendations received by sociodemographic and behavioral characteristics.

Variable		Total Recommendation (0)	Total Recommendation (1–5)	Total Recommendation (6–9)	Total Recommendation (10)
Total		10.65 (10.24-11.06)	20.14 (19.61-20.67)	35.82 (35.19-36.45)	33.39 (32.77-34)
Sex	Female	10.04 (9.51-10.57)	19.22 (18.51-19.92)	37 (36.14-37.85)	33.75 (32.91-34.58)
	Male	11.41 (10.78-12.03)	21.29 (20.48-22.09)	34.37 (33.44-35.3)	32.94 (32.01-33.86)
Age Group	18-39 years old	11.24 (10.58-11.89)	22.36 (21.49-23.24)	34.08 (33.09-35.06)	32.32 (31.35-33.29)
	40-59 years old	10.4 (9.75-11.04)	18.67 (17.84-19.51)	36.36 (35.34-37.37)	34.58 (33.58-35.57)
	≥ 60 years old	10.04 (9.18-10.89)	18.73 (17.61-19.85)	38.06 (36.68-39.45)	33.17 (31.83-34.51)
Residential Area	Rural	9.23 (8.5-9.96)	17.88 (16.91-18.85)	38.1 (36.87-39.34)	34.79 (33.58-36)
	Urban	11.12 (10.64-11.61)	20.9 (20.27-21.53)	35.06 (34.33-35.79)	32.92 (32.2-33.64)
Marriage Status	Never Married	12.04 (10.93-13.14)	23.42 (21.99-24.85)	31.69 (30.12-33.25)	32.85 (31.27-34.44)
	Married	10.29 (9.83-10.74)	19.58 (18.98-20.18)	36.54 (35.81-37.26)	33.6 (32.89-34.31)
	Divorced/Widowed	11.5 (10.03-12.97)	19.4 (17.59-21.2)	36.71 (34.52-38.91)	32.39 (30.26-34.52)
Years of Schooling	illiterate/1–6	10.19 (9.54-10.83)	18.9 (18.06-19.74)	37.63 (36.6-38.66)	33.28 (32.29-34.28)
	7-11	11.04 (10.1-11.98)	19.48 (18.28-20.68)	36.38 (34.92-37.84)	33.1 (31.68-34.52)
	≥12	10.93 (10.3-11.57)	21.53 (20.69-22.37)	34.09 (33.13-35.05)	33.44 (32.49-34.39)
Employment Status	Unemployed	13.1 (11.17-15.02)	23.57 (21.19-25.95)	34.21 (31.55-36.87)	29.13 (26.59-31.66)
	Unpaid Job	9.76 (9.2-10.32)	18.89 (18.14-19.64)	37.54 (36.63-38.46)	33.8 (32.91-34.69)
	Employed	11.47 (10.77-12.16)	21.86 (20.96-22.77)	33.1 (32.07-34.12)	33.57 (32.54-34.6)
	Retired	10.91 (9.56-12.27)	18.04 (16.31-19.77)	38.91 (36.77-41.04)	32.14 (30.1-34.18)
Wealth Index	1^st^ quantile (poorest)	11.68 (10.7-12.66)	22.39 (21.11-23.67)	32.81 (31.39-34.24)	33.12 (31.7-34.54)
	2^nd^ quantile	10.71 (9.79-11.64)	19.92 (18.72-21.13)	34.99 (33.56-36.42)	34.38 (32.95-35.8)
	3^rd^ quantile	10.59 (9.64-11.53)	21 (19.73-22.28)	37.04 (35.57-38.52)	31.36 (29.96-32.77)
	4^th^ quantile	9.96 (9.06-10.87)	19.44 (18.22-20.65)	37.6 (36.13-39.07)	33 (31.58-34.42)
	5^th^ quantile (richest)	10.42 (9.5-11.34)	17.52 (16.39-18.66)	35.53 (34.1-36.96)	36.53 (35.09-37.96)
Insurance Status	None	13.49 (12.02-14.96)	23.59 (21.76-25.43)	30.3 (28.35-32.25)	32.62 (30.6-34.63)
	Basic	10.24 (9.73-10.74)	20.39 (19.72-21.07)	35.37 (34.58-36.17)	34 (33.21-34.79)
	Basic + Complementary	10.76 (9.98-11.54)	18.39 (17.4-19.38)	38.54 (37.31-39.77)	32.31 (31.13-33.48)
BMI	Normal/Underweight	12.17 (11.47-12.87)	21.95 (21.05-22.84)	33.25 (32.24-34.27)	32.63 (31.62-33.63)
	Overweight	10.06 (9.42-10.71)	19.61 (18.75-20.47)	35.95 (34.92-36.97)	34.38 (33.36-35.39)
	Obese	9.32 (8.54-10.1)	18.08 (17.04-19.12)	39.65 (38.35-40.94)	32.95 (31.71-34.19)
Physical Activity	Appropriate	10.31 (9.72-10.91)	19.26 (18.49-20.03)	36.84 (35.9-37.77)	33.59 (32.68-34.51)
	Inappropriate	10.74 (10.14-11.34)	20.96 (20.16-21.76)	34.51 (33.58-35.44)	33.79 (32.87-34.71)
Tobacco Use	Never Smoker	10.86 (10.31-11.41)	18.7 (18-19.39)	35.11 (34.27-35.94)	35.33 (34.5-36.17)
	Passive Smoker	9.57 (8.8-10.34)	20.75 (19.66-21.84)	38.99 (37.69-40.29)	30.69 (29.47-31.91)
	Past Smoker	11.99 (10.15-13.82)	23.1 (20.73-25.47)	39 (36.24-41.76)	25.92 (23.41-28.42)
	Current Smoker	11.1 (9.98-12.22)	23.82 (22.31-25.33)	32.15 (30.49-33.8)	32.93 (31.28-34.59)
Alcohol Use	Never Drinker	10.51 (10.09-10.93)	19.8 (19.25-20.35)	35.72 (35.06-36.37)	33.97 (33.33-34.62)
	Past Drinker	12.79 (10.29-15.29)	24.82 (21.5-28.13)	36.36 (32.66-40.06)	26.03 (22.63-29.44)
	Current Drinker	11.18 (9.02-13.34)	25.37 (22.38-28.35)	38.4 (35.03-41.78)	25.05 (22.05-28.05)

Data are presented as weighted percentages with 95% confidence intervals.

**Fig 1:**
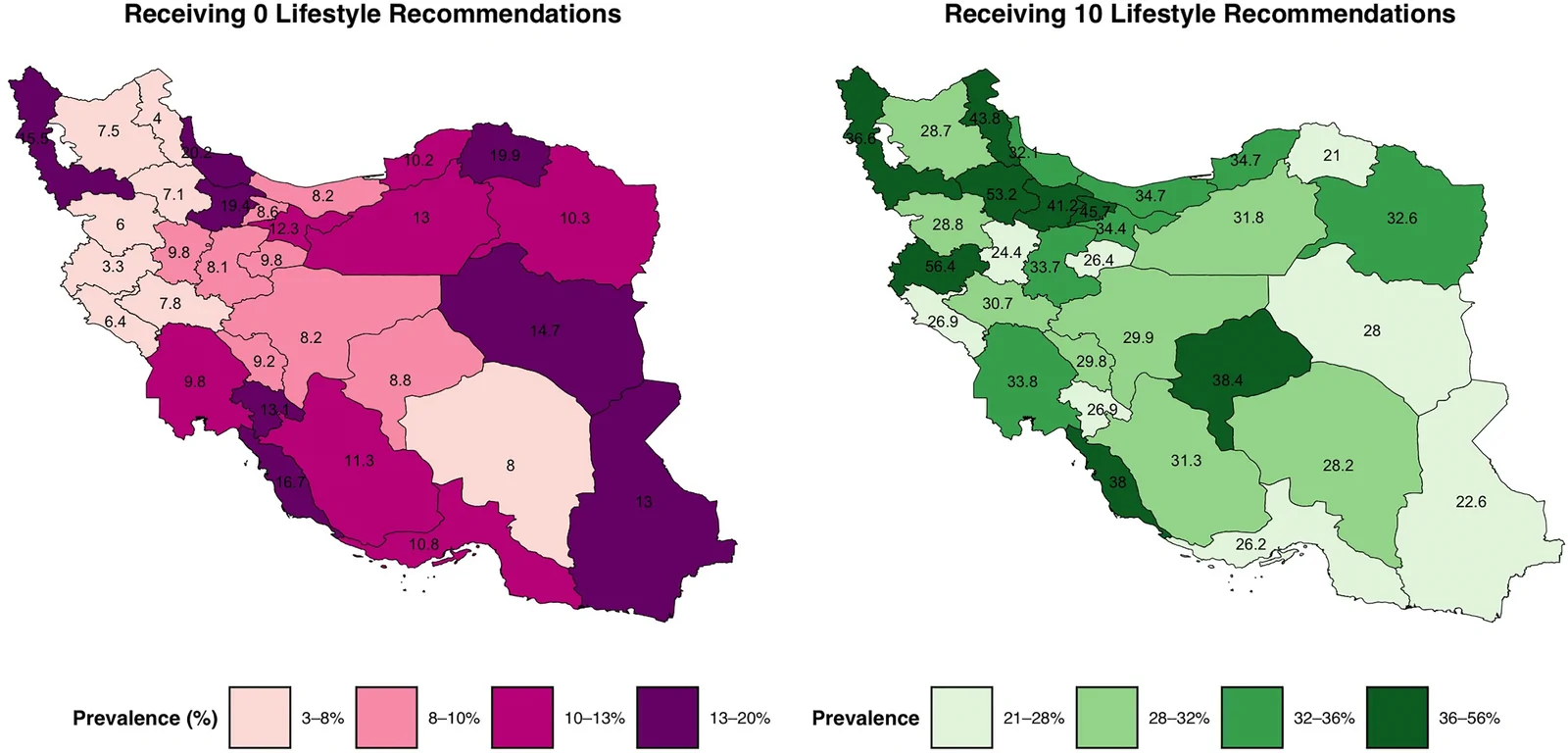
Weighted prevalence of individuals who received all ten recommendations and those who received none by province. Administrative boundaries shown in the map were created in-house by the Non-Communicable Diseases Research Center (NCDRC), Tehran University of Medical Sciences, using ArcGIS software and based on the administrative divisions of Iran. No third-party basemap or external shapefile was used. Study-specific data were visualized by the authors using R.

Most lifestyle recommendations were delivered more frequently to female participants compared to males, with the exception of tobacco non-initiation/cessation, which was more often recommended to males (51.9% [95%CI: 50.9–52.9] among males vs. 45.0% [44.13–45.89] in females). For most other recommendations, sex-specific differences in delivery were unremarkable (S2 Table).

Participants living in rural areas received lifestyle recommendations more often than those in urban settings across dietary, physical activity, weight management, and tobacco categories, while other sociodemographic factors, including age, marital status, education, and employment status, showed recommendation patterns similar to overall sample (Table 2; S2 Table).

### Distribution of lifestyle recommendations across behavioral risk factors

Across BMI categories, lifestyle recommendations were more frequently provided to individuals with higher BMI for most advice types, including dietary and physical activity recommendations, showing a consistent increase from the normal weight to the obese group; the weight-loss/weight-maintenance recommendation showed the most pronounced gradient (62.8% [61.7–63.8] in the normal-weight group to 76.3% [75.1–77.4] in the obese group) (S2 Table). No statistically significant differences were observed between the overweight and obese groups for these recommendations. Among those with insufficient physical activity, the only recommendation delivered significantly more often was exercise advice (74.1%, 73.2–75.0).

Current smokers were significantly more advised to quit smoking (63.4%, 61.6–65.1), yet received other recommendations less frequently than never-smokers. Moreover, tobacco non-initiation/cessation was generally less delivered to all subgroups compared to other recommendations. In terms of alcohol use, current and past alcohol consumers received most recommendations less frequently than non-consumers, with the exception of tobacco non-initiation/cessation advice (S2 Table).

### Distribution of lifestyle recommendations across comorbidity groups

In general, the prevalence of receiving lifestyle recommendations increased with the number of comorbidities, with the exception of tobacco non-initiation/cessation advice, which showed no significant trend (Table 3). The prevalence of individual recommendations by comorbidity group is presented in S3 Table.Participants with diabetes, hypertension, hypercholesterolemia, or CVD received almost all recommendations more frequently than participants without these conditions, except for smoking non-initiation/cessation, which showed no difference. Individuals with cancer were the most likely to receive recommendations to reduce salt intake (81.5, 95%CI: 76.9–86.2), increase fruit and vegetable consumption (83.0,78.5–87.5), and reduce fat intake (82.2, 77.6–86.8). Recommendations to reduce sugar consumption were given most frequently to individuals with diabetes (82.19, 80.63–83.75) (S3 Table). In contrast, recommendations to reduce red meat consumption, increase fish intake, engage in more physical activity, and lose weight/maintain normal weight were distributed relatively evenly across different comorbid groups.

**Table 3:** Distribution of total lifestyle recommendations received by chronic disease status.

Variable		Total Recommendation (0)	Total Recommendation (1–5)	Total Recommendation (6–9)	Total Recommendation (10)
Diabetes Mellitus	No	11.04 (10.6-11.48)	20.66 (20.1-21.23)	35 (34.33-35.66)	33.3 (32.65-33.95)
	Yes	7.36 (6.3-8.43)	15.7 (14.21-17.2)	42.92 (40.9-44.94)	34.01 (32.09-35.94)
Hypertension	No	11.33 (10.85-11.8)	20.85 (20.24-21.46)	34.38 (33.67-35.08)	33.45 (32.75-34.15)
	Yes	8.24 (7.47-9.01)	17.61 (16.53-18.69)	41.04 (39.66-42.43)	33.11 (31.79-34.43)
Hypercholestrolemia	No	11.26 (10.8-11.72)	20.56 (19.97-21.15)	34.54 (33.85-35.23)	33.64 (32.96-34.32)
	Yes	7.84 (7.01-8.68)	18.25 (17.02-19.48)	41.74 (40.2-43.29)	32.17 (30.71-33.63)
Cardiovascular Disease	No	10.79 (10.36-11.21)	20.32 (19.76-20.87)	35.44 (34.78-36.09)	33.46 (32.82-34.1)
	Yes	8.86 (7.48-10.24)	17.82 (15.95-19.69)	40.89 (38.5-43.28)	32.43 (30.16-34.7)
COPD/ Asthma	No	10.74 (10.32-11.16)	20.15 (19.61-20.7)	35.62 (34.97-36.26)	33.49 (32.85-34.12)
	Yes	8.76 (7-10.53)	19.88 (17.37-22.4)	40.83 (37.76-43.9)	30.52 (27.65-33.39)
Cancer	No	10.73 (10.31-11.14)	20.17 (19.64-20.71)	35.85 (35.22-36.49)	33.25 (32.63-33.87)
	Yes	4.73 (2.29-7.18)	17.64 (12.93-22.34)	35.25 (29.54-40.96)	42.38 (36.45-48.32)
Comorbidities	0	11.94 (11.4-12.49)	21.04 (20.36-21.73)	33.2 (32.41-33.98)	33.82 (33.03-34.61)
	1	9.52 (8.68-10.36)	20.13 (18.97-21.3)	38.4 (37.02-39.79)	31.95 (30.62-33.27)
	2 or more	7.3 (6.47-8.13)	16.82 (15.61-18.04)	42.49 (40.89-44.08)	33.39 (31.87-34.91)

Data are presented as weighted percentages with 95% confidence intervals.

### Association of lifestyle recommendations with behavior outcomes

Except for the recommendation to reduce salt intake, the delivery of lifestyle recommendations was associated with positive behavioral status (Table 4). Among current tobacco users, those who received tobacco cessation recommendation were 1.83 times (95% CI: 1.49–2.24) more likely to attempt quitting tobacco. Similarly, receiving recommendations related to physical activity and weight loss was associated with more favorable behavioral outcomes. Individuals who received recommendations to exercise and to lose weight/maintain normal weight were 1.21 times (1.13–1.29) and 1.15 times (1.07–1.22) more likely to engage in sufficient levels of physical activity, respectively. Participants who received 3−5 nutritional recommendations were 1.12 (1.04–1.21) times more likely to have a better nutritional score compared to those who received 0−2 recommendations. Those who received all six nutritional recommendations had 1.32 times higher odds (1.24–1.41) of having a better nutritional score.

**Table 4:** Associations between lifestyle recommendations and behavioral or health outcomes.

Outcome	Exposure (Recommendation)	Category	Crude OR (95% CI)	P value	Model II † OR (95% CI)	P value	Model III ‡ OR (95% CI)	P value
Tobacco quit attempt (among current users)	Cessation Tobacco recommendation	No	Ref					
		Yes	1.81(1.52-2.16)	<0.0001	1.83(1.54-2.19)	<0.0001	1.83(1.49-2.24)	<0.0001
Appropriate physical activity	Exercise recommendation	No	Ref					
		Yes	1.2(1.13-1.27)	<0.0001	1.21(1.14-1.29)	<0.0001	1.21(1.13-1.29)	<0.0001
	Weight loss/ maintaining normal weight recommendation	No	Ref					
		Yes	1.08(1.02-1.15)	0.008	1.14(1.07-1.21)	<0.0001	1.15(1.07-1.22)	<0.0001
Appropriate Daily Salt Use (<5g)	Salt reduction recommendation	No	Ref					
		Yes	1.17(0.87-1.56)	0.301	1.16(0.86-1.55)	0.328	1.16(0.86-1.57)	0.335
Better nutrition score (ordinal)	Nutrition recommendation count	Low (0–2)	Ref					
		Medium (3–5)	1.14 (1.07, 1.22)	0.0001	1.13 (1.06, 1.21)	0.0003	1.12 (1.04, 1.21)	0.003
		High (6)	1.41 (1.32, 1.50)	<0.0001	1.39 (1.31, 1.48)	<0.0001	1.32 (1.24, 1.41)	<0.0001

**^†^** Model II adjusts for age and sex.

**^‡^** Model III adjusts for a set of covariates selected through backward stepwise selection with a p-value threshold of 0.2, specific to each outcome:

**Tobacco quit attempt (among current users)**: adjusted for age, sex, residential area, employment status, wealth index, hypertension, cardiovascular disease (CVD), COPD/asthma, BMI, and physical activity.

**Appropriate physical activity (with exercise recommendation)**: adjusted for age, sex, residential area, marital status, employment status, insurance, diabetes, hypertension, hypercholesterolemia, CVD, cancer, BMI, tobacco use, and alcohol use.

**Appropriate physical activity (with weight loss recommendation)**: adjusted for age, sex, residential area, years of schooling, employment status, wealth index, insurance, diabetes, hypertension, hypercholesterolemia, CVD, BMI, tobacco use, and alcohol use.

**Appropriate daily salt use**: adjusted for age, sex, years of schooling, hypertension, CVD, BMI, physical activity, tobacco use, and alcohol use.

**Better nutrition score (ordinal: poor > moderate > good**): adjusted for age, sex, marital status, years of schooling, employment status, wealth index, health insurance, diabetes, hypercholesterolemia, CVD, COPD/asthma, BMI, and physical activity.

Ordinal logistic regression was used for ordinal outcome (nutrition score), assuming proportional odds.

## Discussion

This study showed disparities in the delivery of lifestyle recommendations across sociodemographic groups, comorbidity, and health behavior status. Our results demonstrated that the more comorbidities an individual has, the prevalence of lifestyle recommendation received increased. The lifestyle recommendations delivered were shown to be associated with favorable behavioral outcomes. Notably, a dose-response relationship was observed between the number of nutritional recommendations received and improvements in dietary quality. Those individuals receiving all nutritional recommendations were 1.32 times more likely to score better at nutrition scoring. Our analysis also revealed a geographic variation in recommendation delivery, with the lowest coverage in eastern provinces and the highest in the northwest provinces of Iran.

Our findings should also be interpreted within an international context. Incomplete delivery of lifestyle counseling by healthcare providers has been documented across multiple countries, although the prevalence of specific recommendations varies by population and healthcare setting.

In the United States, among adults with hypertension, approximately 59% reported receiving advice to exercise, 49% to reduce salt intake, and 42% to lose weight [24]. A more recent secular trend analysis of NHANES data found that, across the general adult population, only 24.8% received advice to reduce sodium intake, 39.9% were advised to exercise, and approximately one-third received advice to lose or control weight, with only modest improvements over time despite a growing NCD burden [25]. In comparison, the corresponding proportions in our study were 72.3%, 71.0%, and 69.1%, respectively. At the global level, a 2023 systematic review and meta-analysis of 40 studies involving nearly 200,000 participants reported a pooled prevalence of physical activity counseling in primary care of only 37.9% (95% CI: 31.2–44.6%), rising to 65.5% among individuals with diabetes and 56.8% among those with overweight or obesity [26]. The 72.3% exercise counseling rate observed in our study compares favorably to this global benchmark, and the pattern of higher counseling rates among higher-risk subgroups mirrors the risk-based targeting observed internationally. While direct comparisons should be interpreted cautiously because of differences in study populations, survey instruments, and healthcare systems, these findings suggest that lifestyle counseling is relatively well integrated into routine care in Iran. Beyond the United States, comparisons with other LMIC settings using the same WHO STEPS instrument are informative. A 2025 study using the 2020 STEPS survey in Cape Verde found that receiving health advice was associated with female sex, older age, and the presence of multiple chronic conditions, patterns strikingly similar to ours [27] This convergence across geographically and culturally distinct LMIC settings suggests that risk-based targeting of lifestyle counseling, prioritizing those with established disease, may reflect a relatively consistent global pattern in primary care, rather than an Iran-specific phenomenon. It also underscores a shared gap: individuals without diagnosed chronic conditions, who may still benefit substantially from preventive counseling, are systematically less likely to receive it across multiple healthcare systems.

In our study, lifestyle recommendations were generally provided more often to females than to males, with the exception of tobacco non-initiation/cessation advice, which was more frequently delivered to men. Women tend to be more proactive in seeking healthcare and discussing health concerns, resulting in greater exposure to preventive advice [28]. Studies show that female patients report more physical symptoms and illness behavior [29], utilize primary care services more often for preventive and diagnostic purposes, and consult providers more frequently for both physical and mental health concerns [30,31] This greater engagement likely increases their opportunities to receive lifestyle recommendations.

Moreover, our observation that individuals with multiple comorbidities receive more health advice may be attributed to the increased frequency of healthcare visits among patients with multiple chronic conditions, providing more opportunities for counseling interactions. A 2020 study conducted across six middle-income countries found that individuals with multiple chronic conditions are more likely to have their health issues accurately identified; however, this does not necessarily lead to improved disease management or control [32].

Our findings reinforce the role of healthcare providers in promoting healthy behaviors. Consistent with global evidence, lifestyle counseling in our study was associated with measurable behavioral gains. Participants who received weight-control or physical-activity advice were more likely to engage in sufficient activity (aOR 1.15 [1.07–1.22] and 1.21 [1.13–1.29], respectively), and smokers counseled to quit were almost twice as likely to attempt cessation (aOR 1.83 [1.49–2.24]). A graded relationship was also observed between the number of nutritional recommendations received and dietary quality (aOR 1.32 [1.24–1.41] for 6 vs ≤ 2 recommendations), echoing the dose-response patterns reported by Patnode et al. (2017) in their JAMA systematic review of behavioral counseling. More recently, a 2025 review published in the International Journal of General Medicine similarly concluded that lifestyle counseling in primary care is effective across multiple domains, nutrition, physical activity, and tobacco cessation, but remains systematically underutilized, particularly in lower-resource settings [33].

Quantitatively, our effect sizes are modest but directionally consistent with U.S. NHANES analyses: Williams et al. (2021) [34] found that adults with hypertension or diabetes who received lifestyle advice had higher odds of losing weight (aOR 1.93 [1.51–2.48]), increasing physical activity (2.02 [1.73–2.37]), and improving diet (e.g., reducing sodium 4.95 [3.93–6.25]). Similarly, Davis-Ajami et al. (2021) [35] reported that prediabetic adults advised by providers were more likely to increase exercise (1.63 [1.12–2.38]) and modify diet (3.00 [1.82–4.96]). Although our population-level odds ratios are smaller, likely reflecting self-reported advice recall and single-time-point measurement, the consistency of direction supports the behavioral relevance of provider counseling. Moreover, causal evidence from an Iranian randomized trial implementing the WHO-PEN 5As model (Amini et al. 2023) [36] demonstrated significant improvements in nutrition and activity behaviors (P < 0.001) and reductions in BMI (P < 0.001), corroborating that structured, skill-based counseling can translate the associations observed in our cross-sectional data into meaningful health outcomes.

The effectiveness of lifestyle counseling depends not only on its frequency but also on how well it is targeted to individuals’ specific risk profiles. Evidence from clinical and population studies indicates that counseling yields greater benefits when directed toward higher-risk groups. For instance, the U.S. Preventive Services Task Force recommends individualized decisions about lifestyle counseling for adults without cardiovascular risk factors because benefits are modest in low-risk populations, whereas stronger evidence supports counseling for adults with obesity or cardiometabolic risk factors [37,38]. Likewise, risk-factor-targeted programs such as the Finnish post-stroke counseling model [39] and the Canadian SeeKD initiative for chronic-kidney-disease prevention show that individualized goal-oriented counseling can improve adherence, weight control, and smoking cessation among at-risk adults [40].

Our findings are consistent with this pattern. Participants who received behavior-specific recommendations were more likely to report healthier behaviors (Table 4), for example, current tobacco users advised to quit were almost twice as likely to attempt cessation (aOR 1.83, 95% CI: 1.49–2.24), and those counseled on weight control or physical activity had greater odds of meeting activity guidelines. Several other results in our study also reflected appropriate risk-based targeting. Across BMI categories, lifestyle recommendations such as reducing fat, sugar, and salt intake and increasing physical activity were more frequently provided to individuals with higher BMI, showing a consistent increase from normal weight to overweight and obese groups. Likewise, participants with a greater number of comorbidities were more likely to receive most types of lifestyle advice, and those with diabetes, hypertension, hypercholesterolemia, or cardiovascular disease were counseled more often than those without these conditions. These trends suggest that healthcare providers partially tailor preventive counseling to individuals’ clinical risk profiles, particularly for obesity and chronic metabolic conditions. However, our findings also revealed important deviations from this ideal pattern. For instance, tobacco counseling remained the least frequently delivered recommendation across all sociodemographic and clinical groups, even among those with multiple comorbidities or higher behavioral risk. Moreover, current smokers and alcohol consumers, despite being high-risk groups, received fewer other lifestyle recommendations. Similarly, while individuals with insufficient physical activity were more likely to be advised to exercise (74.1%, 95% CI: 73.2–75.0), they did not receive other types of lifestyle counseling more frequently, despite being at elevated overall risk. This selective emphasis indicates missed opportunities to reinforce broader preventive behaviors among high-risk individuals. Strengthening the quality and precision of targeting, so that counseling consistently reflects each patient’s risk profile—could substantially enhance the overall effectiveness of preventive care.

When comparing the provincial distribution of lifestyle counseling in this study with the spatial distribution of NCD risk factors and mortality reported by Farzadfar et al.[41] (2024), clear mismatches emerge. When comparing provincial counseling coverage with the quantitative distribution of risk factors reported by Farzadfar et al. (2024), provinces such as Sistan-Baluchestan (salt ≈ 9.2 g/day, obesity ≈ 48% women), Hormozgan (obesity ≈ 51%, diabetes ≈ 10%), and Khuzestan (hypertension ≈ 40%, diabetes ≈ 11%), which represent some of the highest metabolic risk levels in the country, had the lowest proportions of adults receiving all ten lifestyle recommendations (< 30%). In contrast, north-western provinces (Kurdistan, Azerbaijan, Ardabil), where smoking exceeds 40% in men and hypertension > 40%, demonstrated the highest counseling coverage (> 45%), suggesting regional inequities in preventive service distribution.

The observed regional disparities in lifestyle counseling suggest a substantial unmet need for preventive advice. Such inequities reflect persistent gaps in the capacity and distribution of the preventive health workforce across Iran’s provinces. Evidence indicates that primary care physicians in rural and underserved areas often face limited training opportunities in behavioral counseling, as well as time and resource constraints [42,43]. Strengthening capacity building for health personnel—especially among physicians deployed under Iran’s mandatory rural service program—could therefore be a strategic priority. The use of virtual and blended e-learning platforms has proven effective in improving provider competencies, and can be implemented for lifestyle and NCD counseling in primary care settings. Integrating such digital training modules into existing programs like IraPEN and aligning them with WHO’s Package of Essential Noncommunicable Disease Interventions (PEN) [44] could enhance the reach and consistency of lifestyle counseling across provinces. Building a standardized, continuing education framework for preventive counseling within Iran’s PHC network would ensure that all healthcare providers, particularly those entering high-turnover rural posts, receive the skills, tools, and support needed to address patients’ behavioral risks effectively. This approach would help reduce unmet need for lifestyle advice and strengthen progress toward equitable, risk-based NCD prevention nationwide.

A major strength of this study is the use of a large, nationally and subnationally representative dataset, allowing for generalizability of findings across Iran. The comprehensive assessment of various lifestyle recommendations and their associations with both behavior and sociodemographic factors provides insight into preventive care delivery. Moreover, the application of multivariable regression models helps control for potential confounding factors.

However, this study also has limitations. The cross-sectional design limits causal inference, as temporality between receiving recommendations and behavior change cannot be established. Additionally, all behavioral data were self-reported, which may be subject to recall or social desirability bias. Another limitation relates to the categorization of the number of lifestyle recommendations received. Although grouping participants according to the number of recommendations facilitated interpretation and presentation of the findings, categorizing an inherently continuous variable may result in loss of information, reduced statistical power, and potential residual confounding. Furthermore, the selected cut-points are inherently arbitrary and may obscure underlying dose-response relationships [45]. Future studies may benefit from modeling the number of recommendations as a continuous variable or exploring alternative approaches that preserve the full range of information.

Another limitation is the use of backward stepwise regression for covariate selection in the fully adjusted models. Although this approach is commonly used in epidemiological studies to reduce residual confounding, it has well-known drawbacks, including potential model overfitting, instability of selected variables, and inflated Type I error rates. These issues may affect the reproducibility of the findings.

Moreover, the study does not capture the quality or content of lifestyle recommendations, nor the context in which they were delivered, factors that could significantly influence their effectiveness. Finally, the lifestyle recommendations examined were limited to those captured by the WHO STEPS 2021 survey instrument, which substantially overlaps with but does not fully encompass the six pillars of lifestyle medicine [46]. Specifically, recommendations related to restorative sleep, stress management, and positive social connections were not assessed, which may limit the comprehensiveness of our evaluation of lifestyle counseling delivery in Iran.

## Conclusion

This study provides nationally representative evidence on the delivery of lifestyle recommendations by healthcare providers in Iran, revealing both strengths and gaps in the current system of preventive care. Our findings demonstrate that lifestyle advice—particularly regarding diet, physical activity, and weight management—is positively associated with healthier behaviors, with a notable dose-response relationship between the number of nutritional recommendations and diet quality. However, given the cross-sectional design and reliance on self-reported data, causal or temporal conclusions cannot be drawn and the quality or content of the recommendations delivered was not assessed. The pattern of increased advice among individuals with multiple comorbidities suggests a risk-based approach by providers, yet gaps remain—especially in the under-delivery of tobacco non-initiation/cessation counseling to high-risk individuals. Geographic disparities further highlight the unequal reach of health education and intervention across provinces. These findings suggest opportunities for more equitable and consistent delivery of lifestyle counselling within Iran’s national NCD prevention efforts. Strengthening provider training and tailoring strategies to underserved regions may support progress toward these goals, though longitudinal studies are needed to confirm whether such improvements translate into sustained behavioral change and better long-term health outcomes.

## Supporting information

S1 Table Scores of different categories of nutritional variables. (DOCX)

S2 Table Prevalence of individual lifestyle recommendations received by sociodemographic and behavioral characteristics. (DOCX)

S3 Table Prevalence of individual lifestyle recommendations received by chronic disease status. (DOCX)
