# Supplementary material for: Healthcare-delivered lifestyle recommendations and their association with health behaviors in Iran
Source: PLoS One. 2026 Nov 13;21(11):e0345676. doi: 10.1371/journal.pone.0345676 (PMC13766246; doi:10.1371/journal.pone.0345676)
Supplement: Supplementary file 1 [file pone.0345676.s001.docx]

**Supplementary Table 1:** Scores of different categories of nutritional variables

| Variable | Categories | Score |
| --- | --- | --- |
| Mean daily fruit servings* | ≥4 servings | 1 |
|  | 3 ≤ and < 4 servings | 0.75 |
|  | 2 ≤ and < 3 servings | 0.5 |
|  | 1 ≤ and < 2 serving | 0.25 |
|  | <1 servings | 0 |
| Mean daily vegetables servings* | ≥4 servings | 1 |
|  | 3 ≤ and < 4 servings | 0.75 |
|  | 2 ≤ and < 3 servings | 0.5 |
|  | 1 ≤ and < 2 serving | 0.25 |
|  | <1 servings | 0 |
| Frequency of consuming fish | Less than 1 time/month or never | 0 |
|  | 1-3 times/month | 0.2 |
|  | 1-3 times/week | 0.4 |
|  | 4-6 times/week | 0.6 |
|  | 1 or 2 times a day | 0.8 |
|  | More than 3 times a day | 1 |
| Frequency of consuming whole grains | Less than 1 time/month or never | 0 |
|  | 1-3 times/month | 0.2 |
|  | 1-3 times/week | 0.4 |
|  | 4-6 times/week | 0.6 |
|  | 1 or 2 times a day | 0.8 |
|  | More than 3 times a day | 1 |
| Frequency of consuming red meat | Less than 1 time/month or never | 0 |
|  | 1-3 times/month | 0.2 |
|  | 1-3 times/week | 1 |
|  | 4-6 times/week | 0.8 |
|  | 1 or 2 times a day | 0.6 |
|  | More than 3 times a day | 0.4 |
| Frequency of sugar-sweetened beverages consumption | Less than 1 time/month or never | 1 |
|  | 1-3 times/month | 0.8 |
|  | 1-3 times/week | 0.6 |
|  | 4-6 times/week | 0.4 |
|  | 1 or 2 times a day | 0.2 |
|  | More than 3 times a day | 0 |

Mean daily fruit and vegetables serving were calculated by multiplying the reported number of servings consumed per day by the number of days they were consumed in a week, and then dividing the result by 7. This provides an average daily intake over the course of a week.
