# Supplementary material for: Healthcare-delivered lifestyle recommendations and their association with health behaviors in Iran
Source: PLoS One. 2026 Nov 13;21(11):e0345676. doi: 10.1371/journal.pone.0345676 (PMC13766246; doi:10.1371/journal.pone.0345676)
Supplement: Supplementary file 2 [file pone.0345676.s002.docx]

**S2 Table. Prevalence of Individual Lifestyle Recommendations Received by Sociodemographic and Behavioral Characteristics.**

| **Variable** | | **tobacco non-initiation/cessation** | **Decreasing Salt Intake** | **Daily Fruit and Vegetables** | **Reducing Fat consumption** | **Reducing Red Meat and processed food** | **Increasing Fish consumption** | **Whole Grain Bread and Complex Carbs** | **Reducing Sugar** | **Exercise** | **Losing weight/maintaining normal weight** | **Total Recommendation (0)** | **Total Recommendation (1-5)** | **Total Recommendation (6-9)** | **Total Recommendation (10)** |
| --- | --- | --- | --- | --- | --- | --- | --- | --- | --- | --- | --- | --- | --- | --- | --- |
| Total | | 49.81 (49.15-50.46) | 70.99 (70.39-71.59) | 76.99 (76.43-77.55) | 72.46 (71.87-73.04) | 67 (66.38-67.62) | 68.95 (68.34-69.56) | 64.96 (64.33-65.59) | 71.25 (70.66-71.85) | 72.28 (71.69-72.87) | 69.07 (68.46-69.68) | 10.65 (10.24-11.06) | 20.14 (19.61-20.67) | 35.82 (35.19-36.45) | 33.39 (32.77-34) |
| Sex | Female | 45.01 (44.13-45.89) | 72.05 (71.25-72.86) | 78.48 (77.74-79.21) | 74.79 (74.02-75.57) | 68.71 (67.89-69.54) | 70.48 (69.67-71.29) | 66.52 (65.68-67.35) | 72.91 (72.13-73.7) | 73.1 (72.31-73.88) | 71.65 (70.85-72.45) | 10.04 (9.51-10.57) | 19.22 (18.51-19.92) | 37 (36.14-37.85) | 33.75 (32.91-34.58) |
|  | Male | 51.87 (50.89-52.85) | 69.67 (68.77-70.58) | 75.14 (74.29-76) | 69.56 (68.65-70.46) | 64.88 (63.94-65.82) | 67.05 (66.12-67.97) | 63.03 (62.08-63.98) | 69.19 (68.28-70.1) | 71.26 (70.37-72.15) | 65.87 (64.94-66.81) | 11.41 (10.78-12.03) | 21.29 (20.48-22.09) | 34.37 (33.44-35.3) | 32.94 (32.01-33.86) |
| Age Group | 18-39 years old | 48.47 (47.43-49.51) | 67.4 (66.42-68.38) | 76.27 (75.38-77.15) | 69.07 (68.11-70.04) | 64.01 (63-65.01) | 67.03 (66.05-68.01) | 61.44 (60.42-62.45) | 68.17 (67.2-69.14) | 72.26 (71.32-73.19) | 67.73 (66.76-68.71) | 11.24 (10.58-11.89) | 22.36 (21.49-23.24) | 34.08 (33.09-35.06) | 32.32 (31.35-33.29) |
|  | 40-59 years old | 48.35 (47.3-49.4) | 71.92 (70.97-72.87) | 76.81 (75.91-77.71) | 73.97 (73.03-74.9) | 68.74 (67.76-69.72) | 69.93 (68.95-70.9) | 67.08 (66.08-68.08) | 73.01 (72.07-73.95) | 73.38 (72.44-74.33) | 71.01 (70.05-71.98) | 10.4 (9.75-11.04) | 18.67 (17.84-19.51) | 36.36 (35.34-37.37) | 34.58 (33.58-35.57) |
|  | ≥ 60 years old | 46.85 (45.43-48.27) | 75.91 (74.68-77.13) | 78.65 (77.47-79.83) | 75.92 (74.7-77.15) | 69.33 (68.01-70.65) | 70.7 (69.4-72) | 67.57 (66.23-68.91) | 73.72 (72.46-74.97) | 70.28 (68.98-71.58) | 67.97 (66.63-69.31) | 10.04 (9.18-10.89) | 18.73 (17.61-19.85) | 38.06 (36.68-39.45) | 33.17 (31.83-34.51) |
| Residential Area | Rural | 51.02 (49.75-52.29) | 74.92 (73.82-76.02) | 80.67 (79.67-81.66) | 74.98 (73.88-76.08) | 70.09 (68.93-71.25) | 72.24 (71.12-73.37) | 66.77 (65.58-67.96) | 73.04 (71.92-74.16) | 74.42 (73.31-75.52) | 71.43 (70.29-72.58) | 9.23 (8.5-9.96) | 17.88 (16.91-18.85) | 38.1 (36.87-39.34) | 34.79 (33.58-36) |
|  | Urban | 47.09 (46.33-47.86) | 69.68 (68.97-70.39) | 75.76 (75.1-76.42) | 71.61 (70.92-72.31) | 65.97 (65.24-66.7) | 67.85 (67.13-68.57) | 64.35 (63.62-65.09) | 70.65 (69.95-71.35) | 71.56 (70.87-72.26) | 68.28 (67.56-69) | 11.12 (10.64-11.61) | 20.9 (20.27-21.53) | 35.06 (34.33-35.79) | 32.92 (32.2-33.64) |
| Marriage Status | Never Married | 49.13 (47.44-50.81) | 65.78 (64.18-67.39) | 75.94 (74.5-77.38) | 66.2 (64.61-67.8) | 63.01 (61.38-64.63) | 64.77 (63.16-66.38) | 60.74 (59.09-62.38) | 66.42 (64.83-68.02) | 72.67 (71.17-74.17) | 65.96 (64.37-67.56) | 12.04 (10.93-13.14) | 23.42 (21.99-24.85) | 31.69 (30.12-33.25) | 32.85 (31.27-34.44) |
|  | Married | 48.1 (47.35-48.85) | 71.81 (71.13-72.49) | 77.19 (76.56-77.83) | 73.5 (72.84-74.17) | 67.65 (66.94-68.35) | 69.82 (69.13-70.51) | 65.82 (65.1-66.53) | 72.22 (71.55-72.89) | 72.57 (71.89-73.24) | 69.73 (69.03-70.42) | 10.29 (9.83-10.74) | 19.58 (18.98-20.18) | 36.54 (35.81-37.26) | 33.6 (32.89-34.31) |
|  | Divorced/Widowed | 45.95 (43.68-48.22) | 72.82 (70.78-74.85) | 77 (75.05-78.94) | 74.06 (72.05-76.07) | 68.21 (66.08-70.34) | 68.44 (66.31-70.56) | 64.67 (62.49-66.86) | 71.03 (68.95-73.11) | 68.9 (66.77-71.02) | 68.6 (66.47-70.73) | 11.5 (10.03-12.97) | 19.4 (17.59-21.2) | 36.71 (34.52-38.91) | 32.39 (30.26-34.52) |
| Years of Schooling | illiterate/1-6 | 48.42 (47.36-49.49) | 74.47 (73.54-75.4) | 78.02 (77.13-78.91) | 75.01 (74.09-75.94) | 69.01 (68.03-70) | 69.95 (68.98-70.93) | 66.16 (65.15-67.17) | 72.86 (71.91-73.8) | 70.92 (69.96-71.89) | 69.14 (68.15-70.12) | 10.19 (9.54-10.83) | 18.9 (18.06-19.74) | 37.63 (36.6-38.66) | 33.28 (32.29-34.28) |
|  | 7-11 | 50.04 (48.53-51.56) | 70.33 (68.94-71.71) | 76.93 (75.65-78.2) | 71.28 (69.91-72.65) | 66.56 (65.13-67.98) | 68.32 (66.91-69.73) | 64.04 (62.59-65.49) | 70.14 (68.76-71.52) | 72.26 (70.9-73.61) | 69.32 (67.92-70.71) | 11.04 (10.1-11.98) | 19.48 (18.28-20.68) | 36.38 (34.92-37.84) | 33.1 (31.68-34.52) |
|  | ≥12 | 46.8 (45.79-47.81) | 68.23 (67.28-69.17) | 75.98 (75.11-76.85) | 70.7 (69.77-71.62) | 65.33 (64.36-66.3) | 68.21 (67.26-69.15) | 64.12 (63.15-65.09) | 70.19 (69.26-71.12) | 73.38 (72.48-74.28) | 68.75 (67.8-69.69) | 10.93 (10.3-11.57) | 21.53 (20.69-22.37) | 34.09 (33.13-35.05) | 33.44 (32.49-34.39) |
| Employment Status | Unemployed | 49.4 (46.6-52.2) | 64.95 (62.26-67.64) | 73.71 (71.23-76.2) | 66.32 (63.65-68.98) | 62.27 (59.54-64.99) | 62.85 (60.14-65.56) | 58.86 (56.1-61.61) | 65.88 (63.2-68.55) | 65.64 (62.96-68.32) | 61.34 (58.6-64.09) | 13.1 (11.17-15.02) | 23.57 (21.19-25.95) | 34.21 (31.55-36.87) | 29.13 (26.59-31.66) |
|  | Unpaid Job | 45.82 (44.88-46.76) | 72.87 (72.02-73.71) | 79.12 (78.35-79.9) | 74.97 (74.15-75.8) | 68.94 (68.07-69.82) | 70.67 (69.8-71.53) | 66.67 (65.77-67.56) | 73.13 (72.29-73.97) | 73.46 (72.62-74.29) | 71.84 (70.99-72.7) | 9.76 (9.2-10.32) | 18.89 (18.14-19.64) | 37.54 (36.63-38.46) | 33.8 (32.91-34.69) |
|  | Employed | 51.46 (50.37-52.55) | 68.84 (67.83-69.85) | 74.56 (73.61-75.51) | 69.62 (68.62-70.62) | 64.6 (63.56-65.64) | 66.88 (65.85-67.9) | 62.47 (61.42-63.53) | 68.89 (67.88-69.9) | 71.62 (70.64-72.61) | 66.47 (65.44-67.51) | 11.47 (10.77-12.16) | 21.86 (20.96-22.77) | 33.1 (32.07-34.12) | 33.57 (32.54-34.6) |
|  | Retired | 45.12 (42.94-47.3) | 72.81 (70.84-74.77) | 76.66 (74.78-78.54) | 73.67 (71.72-75.62) | 68.4 (66.35-70.46) | 70.92 (68.91-72.93) | 68.38 (66.32-70.43) | 73.09 (71.15-75.03) | 72.18 (70.21-74.14) | 68.58 (66.53-70.63) | 10.91 (9.56-12.27) | 18.04 (16.31-19.77) | 38.91 (36.77-41.04) | 32.14 (30.1-34.18) |
| Wealth Index | 1^st^ quantile (poorest) | 48.8 (47.29-50.31) | 70.23 (68.84-71.62) | 74.79 (73.46-76.12) | 71.43 (70.06-72.81) | 65.79 (64.35-67.23) | 65.35 (63.91-66.8) | 62.39 (60.92-63.86) | 68.61 (67.2-70.02) | 68.85 (67.44-70.26) | 65.34 (63.89-66.79) | 11.68 (10.7-12.66) | 22.39 (21.11-23.67) | 32.81 (31.39-34.24) | 33.12 (31.7-34.54) |
|  | 2^nd^ quantile | 50.24 (48.73-51.74) | 72.15 (70.8-73.5) | 77.38 (76.12-78.65) | 72.62 (71.28-73.96) | 68.14 (66.74-69.54) | 69.38 (67.99-70.76) | 64.76 (63.32-66.2) | 71.11 (69.75-72.46) | 71.51 (70.15-72.87) | 69.6 (68.21-70.98) | 10.71 (9.79-11.64) | 19.92 (18.72-21.13) | 34.99 (33.56-36.42) | 34.38 (32.95-35.8) |
|  | 3^rd^ quantile | 47.6 (46.07-49.12) | 70.47 (69.06-71.87) | 77.18 (75.89-78.47) | 71.53 (70.15-72.92) | 65.74 (64.28-67.21) | 66.68 (65.22-68.13) | 62.88 (61.4-64.36) | 70.35 (68.94-71.75) | 71.06 (69.66-72.47) | 67.65 (66.2-69.1) | 10.59 (9.64-11.53) | 21 (19.73-22.28) | 37.04 (35.57-38.52) | 31.36 (29.96-32.77) |
|  | 4^th^ quantile | 47.76 (46.24-49.27) | 71.27 (69.89-72.65) | 77.3 (76.02-78.58) | 72.82 (71.46-74.18) | 66.96 (65.53-68.4) | 70.01 (68.61-71.41) | 66.1 (64.66-67.54) | 72.25 (70.9-73.61) | 73.71 (72.38-75.05) | 69.8 (68.4-71.2) | 9.96 (9.06-10.87) | 19.44 (18.22-20.65) | 37.6 (36.13-39.07) | 33 (31.58-34.42) |
|  | 5^th^ quantile (richest) | 48.42 (46.93-49.91) | 71.74 (70.39-73.09) | 78.31 (77.07-79.55) | 74.13 (72.82-75.44) | 68.93 (67.55-70.32) | 72.92 (71.59-74.25) | 68.65 (67.26-70.04) | 73.81 (72.49-75.12) | 76.02 (74.73-77.3) | 72.42 (71.08-73.76) | 10.42 (9.5-11.34) | 17.52 (16.39-18.66) | 35.53 (34.1-36.96) | 36.53 (35.09-37.96) |
| Insurance Status | None | 49.8 (47.66-51.95) | 66.16 (64.12-68.19) | 73.52 (71.62-75.42) | 66.55 (64.52-68.58) | 62.2 (60.12-64.27) | 63.29 (61.23-65.35) | 59.54 (57.44-61.64) | 64.77 (62.71-66.82) | 68.12 (66.12-70.13) | 65.18 (63.14-67.23) | 13.49 (12.02-14.96) | 23.59 (21.76-25.43) | 30.3 (28.35-32.25) | 32.62 (30.6-34.63) |
|  | Basic | 49.25 (48.42-50.08) | 71.47 (70.72-72.23) | 77.39 (76.69-78.09) | 72.62 (71.88-73.37) | 67 (66.22-67.79) | 68.91 (68.14-69.68) | 64.79 (63.99-65.58) | 71.47 (70.72-72.22) | 72.5 (71.76-73.25) | 69.17 (68.39-69.94) | 10.24 (9.73-10.74) | 20.39 (19.72-21.07) | 35.37 (34.58-36.17) | 34 (33.21-34.79) |
|  | Basic + Complementary | 44.72 (43.47-45.97) | 71.49 (70.35-72.64) | 77.07 (75.99-78.14) | 74.04 (72.93-75.15) | 68.4 (67.23-69.58) | 70.74 (69.58-71.89) | 66.93 (65.74-68.12) | 72.75 (71.63-73.87) | 73.04 (71.92-74.17) | 69.92 (68.76-71.09) | 10.76 (9.98-11.54) | 18.39 (17.4-19.38) | 38.54 (37.31-39.77) | 32.31 (31.13-33.48) |
| BMI | Normal/Underweight | 49.52 (48.45-50.6) | 68.43 (67.43-69.44) | 75.85 (74.93-76.78) | 68.19 (67.19-69.2) | 64.08 (63.05-65.11) | 66.91 (65.9-67.93) | 62.6 (61.56-63.64) | 67.97 (66.97-68.97) | 69.98 (68.99-70.96) | 62.75 (61.71-63.8) | 12.17 (11.47-12.87) | 21.95 (21.05-22.84) | 33.25 (32.24-34.27) | 32.63 (31.62-33.63) |
|  | Overweight | 48.29 (47.22-49.36) | 71.95 (70.98-72.91) | 77.59 (76.7-78.49) | 74.33 (73.4-75.27) | 68.07 (67.07-69.08) | 70.01 (69.03-70.99) | 65.94 (64.92-66.96) | 72.44 (71.47-73.4) | 73.01 (72.06-73.97) | 70.47 (69.49-71.45) | 10.06 (9.42-10.71) | 19.61 (18.75-20.47) | 35.95 (34.92-36.97) | 34.38 (33.36-35.39) |
|  | Obese | 45.62 (44.3-46.94) | 73.35 (72.16-74.53) | 77.76 (76.64-78.88) | 76.01 (74.86-77.15) | 69.77 (68.55-71) | 70.3 (69.08-71.52) | 67.06 (65.81-68.31) | 74.33 (73.16-75.49) | 74.57 (73.41-75.74) | 76.27 (75.12-77.41) | 9.32 (8.54-10.1) | 18.08 (17.04-19.12) | 39.65 (38.35-40.94) | 32.95 (31.71-34.19) |
| Physical Activity | Appropriate | 49.12 (48.15-50.09) | 71.47 (70.58-72.35) | 77.62 (76.8-78.43) | 73.06 (72.19-73.92) | 67.42 (66.5-68.33) | 69.61 (68.71-70.51) | 65.45 (64.52-66.38) | 71.59 (70.72-72.47) | 74.06 (73.21-74.91) | 70.18 (69.29-71.07) | 10.31 (9.72-10.91) | 19.26 (18.49-20.03) | 36.84 (35.9-37.77) | 33.59 (32.68-34.51) |
|  | Inappropriate | 46.85 (45.88-47.82) | 70.9 (70.01-71.79) | 76.75 (75.92-77.57) | 72.58 (71.71-73.45) | 66.96 (66.04-67.88) | 68.73 (67.82-69.63) | 64.95 (64.02-65.88) | 71.47 (70.59-72.35) | 70.45 (69.56-71.35) | 68.46 (67.56-69.37) | 10.74 (10.14-11.34) | 20.96 (20.16-21.76) | 34.51 (33.58-35.44) | 33.79 (32.87-34.71) |
| Tobacco Use | Never Smoker | 46.11 (45.23-46.98) | 72.14 (71.35-72.93) | 77.85 (77.11-78.59) | 74.12 (73.35-74.9) | 68.62 (67.8-69.44) | 70.38 (69.57-71.18) | 67.22 (66.39-68.04) | 72.71 (71.93-73.49) | 73.18 (72.4-73.96) | 70.37 (69.57-71.18) | 10.86 (10.31-11.41) | 18.7 (18-19.39) | 35.11 (34.27-35.94) | 35.33 (34.5-36.17) |
|  | Passive Smoker | 43.76 (42.45-45.08) | 71.26 (70.05-72.47) | 78.11 (77.01-79.21) | 72.64 (71.45-73.82) | 67.06 (65.81-68.31) | 69.4 (68.17-70.62) | 64.34 (63.06-65.61) | 71.42 (70.22-72.62) | 73.29 (72.11-74.47) | 70.81 (69.6-72.02) | 9.57 (8.8-10.34) | 20.75 (19.66-21.84) | 38.99 (37.69-40.29) | 30.69 (29.47-31.91) |
|  | Past Smoker | 48.16 (45.33-51) | 67.15 (64.49-69.8) | 73.5 (71-75.99) | 69.28 (66.67-71.89) | 62.27 (59.53-65.01) | 64.48 (61.79-67.18) | 58.25 (55.46-61.04) | 68.05 (65.42-70.67) | 68.7 (66.09-71.31) | 61.12 (58.36-63.89) | 11.99 (10.15-13.82) | 23.1 (20.73-25.47) | 39 (36.24-41.76) | 25.92 (23.41-28.42) |
|  | Current Smoker | 63.35 (61.64-65.06) | 67.42 (65.77-69.08) | 72.96 (71.39-74.54) | 66.72 (65.05-68.39) | 62.1 (60.38-63.81) | 64.14 (62.44-65.84) | 59.49 (57.75-61.22) | 66.32 (64.65-67.99) | 68.34 (66.69-69.99) | 64.03 (62.33-65.73) | 11.1 (9.98-12.22) | 23.82 (22.31-25.33) | 32.15 (30.49-33.8) | 32.93 (31.28-34.59) |
| Alcohol Use | Never Drinker | 47.55 (46.86-48.23) | 71.48 (70.86-72.1) | 77.48 (76.91-78.06) | 73.12 (72.51-73.73) | 67.59 (66.94-68.23) | 69.42 (68.79-70.05) | 65.7 (65.05-66.35) | 71.81 (71.2-72.43) | 72.62 (72.01-73.24) | 69.62 (68.99-70.25) | 10.51 (10.09-10.93) | 19.8 (19.25-20.35) | 35.72 (35.06-36.37) | 33.97 (33.33-34.62) |
|  | Past Drinker | 56.75 (52.95-60.55) | 67.98 (64.44-71.51) | 69.4 (65.88-72.93) | 65.74 (62.13-69.36) | 58.67 (54.89-62.45) | 62.81 (59.11-66.51) | 52.3 (48.47-56.14) | 64.84 (61.19-68.48) | 66.13 (62.52-69.74) | 61.03 (57.3-64.77) | 12.79 (10.29-15.29) | 24.82 (21.5-28.13) | 36.36 (32.66-40.06) | 26.03 (22.63-29.44) |
|  | Current Drinker | 54.04 (50.59-57.48) | 62.32 (58.97-65.67) | 72.07 (68.98-75.15) | 62.96 (59.63-66.29) | 60.36 (57-63.73) | 63.75 (60.44-67.06) | 57.19 (53.78-60.6) | 64.04 (60.75-67.34) | 68.73 (65.55-71.92) | 62.65 (59.33-65.98) | 11.18 (9.02-13.34) | 25.37 (22.38-28.35) | 38.4 (35.03-41.78) | 25.05 (22.05-28.05) |
