# Supplementary material for: Healthcare-delivered lifestyle recommendations and their association with health behaviors in Iran
Source: PLoS One. 2026 Nov 13;21(11):e0345676. doi: 10.1371/journal.pone.0345676 (PMC13766246; doi:10.1371/journal.pone.0345676)
Supplement: Supplementary file 3 [file pone.0345676.s003.docx]

**S3 Table. Prevalence of Individual Lifestyle Recommendations Received by Chronic Disease Status.**

| **Variable** | | **tobacco non-initiation/cessation** | **Decreasing Salt Intake** | **Daily Fruit and Vegetable** | **Reducing Fat consumption** | **Reducing Red Meat and processed food** | **Increasing Fish consumption** | **Whole Grain Bread and Complex Carbs** | **Reducing Sugar** | **Exercise** | **Losing weight/maintaining normal weight** | **Total Recommendation (0)** | **Total Recommendation (1-5)** | **Total Recommendation (6-9)** | **Total Recommendation (10)** |
| --- | --- | --- | --- | --- | --- | --- | --- | --- | --- | --- | --- | --- | --- | --- | --- |
| Diabetes Mellitus | No | 48.26 (47.57-48.96) | 70.25 (69.61-70.89) | 76.64 (76.05-77.23) | 71.71 (71.08-72.34) | 66.32 (65.66-66.98) | 68.42 (67.77-69.06) | 64.17 (63.5-64.83) | 69.96 (69.32-70.6) | 71.64 (71.01-72.27) | 68.27 (67.62-68.92) | 11.04 (10.6-11.48) | 20.66 (20.1-21.23) | 35 (34.33-35.66) | 33.3 (32.65-33.95) |
|  | Yes | 46.42 (44.39-48.45) | 77.26 (75.54-78.97) | 79.96 (78.31-81.6) | 78.77 (77.09-80.45) | 72.74 (70.92-74.57) | 73.39 (71.59-75.2) | 71.65 (69.81-73.49) | 82.19 (80.63-83.75) | 77.65 (75.95-79.35) | 75.85 (74.1-77.6) | 7.36 (6.3-8.43) | 15.7 (14.21-17.2) | 42.92 (40.9-44.94) | 34.01 (32.09-35.94) |
| Hypertension | No | 48.47 (47.73-49.22) | 69.04 (68.35-69.73) | 76.03 (75.39-76.67) | 70.85 (70.17-71.52) | 65.93 (65.22-66.63) | 68.02 (67.32-68.71) | 63.92 (63.2-64.63) | 69.9 (69.22-70.58) | 71.59 (70.92-72.27) | 67.91 (67.21-68.61) | 11.33 (10.85-11.8) | 20.85 (20.24-21.46) | 34.38 (33.67-35.08) | 33.45 (32.75-34.15) |
|  | Yes | 46.62 (45.22-48.02) | 77.98 (76.81-79.15) | 80.41 (79.28-81.53) | 78.21 (77.05-79.38) | 70.79 (69.5-72.08) | 72.27 (71-73.53) | 68.63 (67.33-69.94) | 76.09 (74.88-77.29) | 74.68 (73.45-75.9) | 73.21 (71.96-74.45) | 8.24 (7.47-9.01) | 17.61 (16.53-18.69) | 41.04 (39.66-42.43) | 33.11 (31.79-34.43) |
| Hypercholestrolemia | No | 48.67 (47.94-49.39) | 70.2 (69.53-70.86) | 76.6 (75.98-77.22) | 70.98 (70.32-71.64) | 66.06 (65.37-66.74) | 68.19 (67.51-68.87) | 64.19 (63.5-64.89) | 70.14 (69.48-70.81) | 71.64 (70.98-72.29) | 68.14 (67.46-68.82) | 11.26 (10.8-11.72) | 20.56 (19.97-21.15) | 34.54 (33.85-35.23) | 33.64 (32.96-34.32) |
|  | Yes | 45.29 (43.73-46.85) | 74.63 (73.26-76) | 78.76 (77.46-80.05) | 79.25 (77.97-80.53) | 71.35 (69.92-72.77) | 72.41 (71.01-73.82) | 68.48 (67.02-69.94) | 76.34 (75.01-77.67) | 75.19 (73.84-76.54) | 73.35 (71.96-74.74) | 7.84 (7.01-8.68) | 18.25 (17.02-19.48) | 41.74 (40.2-43.29) | 32.17 (30.71-33.63) |
| Cardiovascular Disease | No | 48.02 (47.34-48.71) | 70.58 (69.96-71.21) | 76.78 (76.2-77.36) | 72.07 (71.45-72.68) | 66.67 (66.03-67.32) | 68.75 (68.12-69.39) | 64.69 (64.03-65.34) | 70.98 (70.36-71.6) | 72.27 (71.66-72.89) | 68.92 (68.28-69.55) | 10.79 (10.36-11.21) | 20.32 (19.76-20.87) | 35.44 (34.78-36.09) | 33.46 (32.82-34.1) |
|  | Yes | 48.69 (46.26-51.12) | 76.22 (74.16-78.28) | 79.91 (77.96-81.86) | 77.48 (75.43-79.52) | 71.12 (68.91-73.33) | 71.51 (69.31-73.71) | 68.5 (66.24-70.76) | 74.74 (72.62-76.86) | 72.52 (70.36-74.69) | 71.1 (68.9-73.3) | 8.86 (7.48-10.24) | 17.82 (15.95-19.69) | 40.89 (38.5-43.28) | 32.43 (30.16-34.7) |
| COPD/ Asthma | No | 48.11 (47.44-48.79) | 70.93 (70.31-71.54) | 76.96 (76.39-77.53) | 72.32 (71.72-72.93) | 66.85 (66.22-67.49) | 68.83 (68.21-69.46) | 64.82 (64.18-65.47) | 71.17 (70.56-71.78) | 72.2 (71.6-72.81) | 68.91 (68.28-69.53) | 10.74 (10.32-11.16) | 20.15 (19.61-20.7) | 35.62 (34.97-36.26) | 33.49 (32.85-34.12) |
|  | Yes | 46.97 (43.85-50.09) | 72.44 (69.65-75.23) | 77.62 (75.01-80.23) | 75.24 (72.54-77.94) | 70.01 (67.15-72.88) | 71.18 (68.36-74) | 67.55 (64.61-70.48) | 72.99 (70.21-75.77) | 73.56 (70.8-76.32) | 72.38 (69.59-75.17) | 8.76 (7-10.53) | 19.88 (17.37-22.4) | 40.83 (37.76-43.9) | 30.52 (27.65-33.39) |
| Cancer | No | 47.94 (47.28-48.6) | 70.87 (70.26-71.47) | 76.92 (76.35-77.48) | 72.33 (71.74-72.93) | 66.88 (66.26-67.51) | 68.81 (68.2-69.43) | 64.83 (64.2-65.47) | 71.19 (70.59-71.79) | 72.24 (71.64-72.83) | 68.98 (68.37-69.6) | 10.73 (10.31-11.14) | 20.17 (19.64-20.71) | 35.85 (35.22-36.49) | 33.25 (32.63-33.87) |
|  | Yes | 58.18 (52.26-64.1) | 81.52 (76.86-86.18) | 83.03 (78.53-87.53) | 82.17 (77.56-86.78) | 76.07 (70.96-81.19) | 79.19 (74.31-84.07) | 74.07 (68.87-79.28) | 76.06 (70.91-81.21) | 74.45 (69.16-79.75) | 75.4 (70.21-80.58) | 4.73 (2.29-7.18) | 17.64 (12.93-22.34) | 35.25 (29.54-40.96) | 42.38 (36.45-48.32) |
| Comorbidities | 0 | 49.16 (48.33-50) | 68.52 (67.74-69.3) | 75.82 (75.1-76.54) | 69.56 (68.79-70.33) | 65.09 (64.29-65.88) | 67.3 (66.51-68.08) | 63.25 (62.44-64.05) | 68.93 (68.16-69.71) | 71.11 (70.35-71.87) | 67.07 (66.28-67.86) | 11.94 (11.4-12.49) | 21.04 (20.36-21.73) | 33.2 (32.41-33.98) | 33.82 (33.03-34.61) |
|  | 1 | 45.47 (44.05-46.89) | 72.76 (71.48-74.04) | 77.75 (76.55-78.95) | 75.11 (73.86-76.35) | 67.84 (66.49-69.18) | 70.17 (68.85-71.49) | 65.57 (64.2-66.93) | 72.36 (71.08-73.64) | 72.72 (71.45-73.99) | 70.45 (69.14-71.76) | 9.52 (8.68-10.36) | 20.13 (18.97-21.3) | 38.4 (37.02-39.79) | 31.95 (30.62-33.27) |
|  | 2 or more | 47.24 (45.63-48.85) | 77.9 (76.56-79.24) | 80.42 (79.14-81.71) | 79.78 (78.48-81.08) | 72.96 (71.52-74.4) | 73.49 (72.07-74.91) | 70.43 (68.96-71.9) | 78.41 (77.09-79.74) | 75.95 (74.57-77.33) | 74.72 (73.31-76.12) | 7.3 (6.47-8.13) | 16.82 (15.61-18.04) | 42.49 (40.89-44.08) | 33.39 (31.87-34.91) |

Data are presented as weighted percentages with 95% confidence intervals.
